# Sensitivity of urinary pathogens for patients discharged from the emergency department compared with the hospital antibiogram

**DOI:** 10.1186/s12873-019-0264-z

**Published:** 2019-09-05

**Authors:** Sean Carlsen, Scott P. Krall, K. Tom Xu, Alainya Tomanec, Daylon Farias, Peter Richman

**Affiliations:** 1 Department of Emergency Medicine, CHRISTUS Health/Texas A&M Health Science Center, 600 Elizabeth Street, Corpus Christi, TX 78404 USA; 2 Department of Family & Community Medicine and Department of Surgery, Texas Tech University Health Sciences Center, School of Medicine, Lubbock, TX 79430 USA

**Keywords:** Diabetic, UTI, Urinary tract infection, Antibiotic sensitivity, Antibiotic resistance, Antibiogram

## Abstract

**Background:**

Data for hospital antibiograms are typically compiled from all patients, regardless of disposition, demographics and other comorbidities.

**Objective:**

We hypothesized that the sensitivity patterns for urinary pathogens would differ significantly from the hospital antibiogram in patients that were discharged from the emergency department (ED).

**Methods:**

We evaluated a retrospective cohort of all adult patients with positive urine cultures treated in the 2016 calendar year at an inner-city academic ED. Positive urine cultures defined by our institution’s microbiology department. Investigators conducted a structured review of an electronic medical record (EMR) to collect demographic, historical and microbiology records. We utilized a one-sample test of proportion to compare the sensitivity of each organism for discharged patients to the hospital published antibiogram. Alpha set at 0.05.

**Results:**

During the study period, 414 patients were discharged from the ED and found to have positive urine cultures; 20% age > 60 years old, 85% female, 79% Hispanic, 33% diabetic. The most common organisms was *E. coli* (78%). *E. coli* was sensitive to Trimethoprim-Sulfamethoxazole for 59% vs. 58% in our antibiogram (*p* = 0.77), Ciprofloxacin 81% vs. 69% (*p* < 0. 001), Nitrofurantoin 96% vs 95%; (*p* = 0.25). *K. pneumoniae* was sensitive to Trimethoprim-Sulfamethoxazole 87% vs. 80% in our antibiogram (*p* = 0.26), Ciprofloxacin 100% vs. 92% (*p* = 0.077), Nitrofurantoin 86% vs 41% (*p* < 0.001).

**Conclusions:**

For our predominantly Hispanic study group with a high prevalence of diabetes, we found that our hospital antibiogram had relatively good value in guiding antibiotic therapy though for some organism/antibiotic combinations sensitivities were higher than expected.

## Background

Urinary tract infections (UTIs) account for approximately 1 million annual ED visits, representing one of the most common infections for which emergency physicians prescribe antibiotics [1]. Yet, unlike other causes for bacterial infections, prescribing habits for uncomplicated UTIs are generally dictated by local hospital antibiograms, varying greatly between certain patient populations and geographic locations. Despite such focused therapy, 12–16% of patients will become treatment failures, adding $1.6 billion dollars annually to any already heavily burdened healthcare system [2].

In our predominately Hispanic-populated inner city with a prevalence of diabetes reaching four times the national average, we have traditionally seen resistance rates to *Trimethoprim-Sulfamethoxazole* and *Ciprofloxacin* well above national averages. This has led to a near complete cessation of prescribing these drugs for uncomplicated UTIs in our hospital over the past several years. Current studies show that diabetics have increased risk for urinary tract infection (UTI) may have increased resistance rates in urinary pathogens [3–5]. Within this context, we hypothesized that we would observe significant differences between the antimicrobial susceptibilities of pathogens obtain from patients diagnosed with UTI then discharged from our ED compared with the published the hospital-wide antibiogram.

## Methods

### Study design

We conducted a retrospective, cross-sectional study to evaluate differences in antimicrobial susceptibility patterns between discharged ED patients and our hospital-wide antibiogram.

### Setting

The study was conducted at CHRISTUS Spohn Hospital System. The facility is a major teaching affiliate of Texas A&M Health Science Center, a level-two trauma center, and serves an inner-city population. The annual ED census is approximately 45,000 patients. The CHRISTUS Health Institutional Review Board approved this study prior to the initiation of data collection.

### Population

Our study included all adult ED patients that had positive urine cultures in 2016.

### Study protocol

The primary investigator and a trained research associate conducted a structured electronic chart review on all patients that had positive urine cultures ordered by the emergency physician identified by our laboratory information system. Patient characteristics that were collected included, ED disposition, gender, ethnicity, age (indicating if over 60 years), presence or absence of diabetes, recent hospitalizations, and insurance status. Next, antimicrobial sensitivity patterns were recorded for all patients with *E. coli* or *K. pneumoniae* bacteria and their susceptibility to Trimethoprim-Sulfamethoxazole, Ciprofloxacin and Nitrofurantoin.

### Statistical analysis

We compared observe pathogen sensitivities to those published on the hospital antibiogram by z-score tests. Alpha was set at 0.05.

## Results

We reviewed the charts of 495 ED patients with positive urine cultures during 2016. Table 1 summarizes the characteristics of these patients; 414 were discharged from the ED (study group). *E coli*. Accounted for nearly 78% of all urinary pathogens in our study group, while *K. pneumoniae* accounted for over 10%. The remaining bacteria isolates included Enterobacter, Enterococcus, Proteus, Pseudomonas, MSSA, MRSA and Citrobacter, but were not included in our analysis because their sample sizes.

**Table 1:** Study Group Characteristics

Age 18–59	80%
Female gender	85%
Hispanic ethnicity	79%
Diabetic	33%
Private insured status	12%
Recent hospitalization	10%

Table 2 summarizes the sensitivity of E Coli in the culture results of our study group to several antibiotics. We found that E Coli sensitivity to Trimethoprim-Sulfamethoxazole and Nitrofurantoin respectively were not significantly different between cultures of discharged patients and the hospital antibiogram. However, we found that sensitivity to *E. coli* from cultured discharged patients was significantly higher to Ciprofloxacin as compared to the hospital antibiogram (81% vs. 69%; z = 4.86; *p* < 0.01).

**Table 2:** Comparison of antimicrobial sensitivity patterns of *E. coli*

	Trimethoprim-Sulfamethoxazole (*n* = 330)	Ciprofloxacin (*n* = 328)	Nitrofurantoin (*n* = 308)
Sensitivity from hospital antibiogram	58%	69%	**95%**
Sensitivity from sample (std) [95% CI]	58.8% (2.7%) [53.4–64.1%]	81.4% (2.1%) [77.2–85.6%]	96.4% (1.0%) [94.4–98.5%]
z-score	.2900	4.8567	1.1504
*p*-value	0.77	0.00	0.25

Table 3 summarizes the sensitivity patterns of *K. pneumoniae* in the culture results of our study group to several antibiotics as compared with the antibiogram. We found no significant difference for cultured sensitivities to Trimethoprim-Sulfamethoxazole and Ciprofloxacin respectively. However, we did find a statistically significant increase in sensitivity of cultured organisms to Nitrofurantoin from patients discharged from the ED when compared to the hospital-wide antibiogram (86% vs. 41%; z = 5.38; *p* < 0.01).

**Table 3:** Comparison of antimicrobial sensitivity patterns of *K. pneumoniae*

	Trimethoprim-Sulfamethoxazole (*n* = 39)	Ciprofloxacin (*n* = 36)	Nitrofurantoin (*n* = 35)
Sensitivity from hospital antibiogram	80%	92%	41%
Sensitivity from sample (std) [95% CI]	87.2% (5.3%) [76.7–97.7%]	100% (0%) [100–100%]	85.7% (5.9%) [74.1–97.3%]
z-score	1.1209	1.7693	5.3785
*p*-value	0.2623	0.0768	0.0000

## Discussion

Several recent studies in prominent emergency medicine literature compared the antimicrobial sensitivity patterns of discharged ED patients and their facility-based antibiograms, however, the respective findings have been far from unifying [6–8]. A retrospective chart review performed by Drapkin, et al. conducted a retrospective review for patients in a university ED setting from 2011 to 2012 and evaluated all urine cultures with greater than 100,000 cfu/mL. The authors found that susceptibility rates in uncomplicated cystitis presenting to the ED were no different than those reported in the hospital-wide antibiogram. This review included 153 *E. coli* samples, and they found the following susceptibilities of Trimethoprim-Sulfamethoxazole (77%), Nitrofurantoin (99%), Ciprofloxacin (84%) and Levofloxacin (85%) [6]. It should be noted that the sensitivity ratios seen in this study population do not correlate with our very low antibiogram sensitivities for Trimethoprim-Sulfamethoxazole of 58% or Ciprofloxacin at 69%. We suspect that comorbid factors, such as an unusually high prevalence of diabetes and renal insufficiency in our may play a role in the differences observed in our study.

In a similar investigation by Smith et al., the authors reviewed 349 urine samples with an abnormality yielding a reflex culture for ED patients. Without examining the specific pathogens, they found greater susceptibility for the cultured organisms for the ED patients as compared to that observed for the hospital antibiogram, including: Trimethoprim-Sulfamethoxazole (80% vs. 71%; *p* < 0.05), Ancef (97% vs. 87%; *p* < 0.05) and Ciprofloxacin (89% vs. 73%; *p* < 0.05) [7]. This study is notable in contrast to ours for additionally comparing the susceptibilities of the organisms cultured from ED patients to an ED-specific antibiogram. For this comparison, the sensitivities of ED cultured organisms were significantly higher for each antibiotic than that observed on the hospital based antibiogram.

Within our study group, we did not distinguish between patients with complicated and uncomplicated UTI. In contrast, Hines, et al. performed a smaller, prospective study that looked at 45 females with uncomplicated symptomatic dysuria or pyuria and found the following differences in susceptibilities between those patients discharged from the ED and their antibiogram: Trimethoprim-Sulfamethoxazole (84% vs. 67%; *p* = 0.016), Levofloxacin (98% vs. 74%; *p* < 0.001), Ciprofloxacin (98% vs. 58%; *p* < 0.001) [8]. We believe larger investigations are warranted to confirm the findings of the report by Hines et al. as it has significant implications for the treatment of uncomplicated UTI in the ED.

While there was significant prior research in this area before we initiated our investigation, we felt that our population in Southern Texas represented an excellent opportunity to evaluate sensitivity patterns in a study group with an unusually high prevalence of Hispanics (79%), many of who suffer from diabetes (33% of the study group patients). Our published rates of Trimethoprim-Sulfamethoxazole and Ciprofloxacin sensitivities were significantly lower than other studies we examined. This suggests that regional/population level patient characteristics may influence antimicrobial sensitivity patterns. We observed within our study population that for *E. coli*, Trimethoprim-Sulfamethoxazole remains a poor choice for uncomplicated cystitis. However, despite higher risk of resistance than that reported in other regions and, nonetheless, consistent with other studies, Ciprofloxacin sensitivity was greater in our ED discharged patients than published in our hospital antibiogram. Finally, our study also appears novel for the evaluation of ED related UTI secondary to *K. pneumonia.* Within our study group, we found significantly improved sensitivity to Nitrofurantoin in patients discharged from the ED vs. our antibiogram.

### Limitations and future questions

Our study has some limitations that warrant discussion. A prospective study would have been more ideal to better define patient subgroups to refine our analysis. For example, our results would have more specific implications if we identified patients by pre-study criteria as complicated vs. uncomplicated UTI.

Our analysis also seems remiss for the absence of an evaluation of the sensitivity patterns for cephalosporins. This was unavoidable due to the testing patterns in our hospital microbiology department with respect to inconsistency in the class of cephalosporin utilized to determine resistance. In the face of increasing overall antibiotic resistance, future studies should evaluate the sensitivity of ED-based urine culture organisms to off-patent first generation cephalosporins as well as the more expensive higher generations.

## Conclusions

In conclusion, for our predominantly Hispanic study group with a high prevalence of diabetes, we found that our hospital antibiogram had relatively good value in guiding antibiotic therapy. We did find a significantly higher sensitivity of *E. coli* to ciprofloxacin and likewise, in *K. pneumoniae* to Nitrofurantoin. Our results are consistent with other studies that suggest that Trimethoprim-Sulfamethoxazole remains a poor choice in UTI.

## Data Availability

The datasets used and/or analyzed during the current study are available from the corresponding author on reasonable request.

## Abbreviations

ED: Emergency department
EMR: Electronic medical record
UTI: Urinary tract infection
UTIs: URINARY tract infections
